# Spontaneous Pneumomediastinum and Subcutaneous Emphysema: A Rare Complication Associated With Cocaine Consumption

**DOI:** 10.7759/cureus.70826

**Published:** 2024-10-04

**Authors:** Sergio L Jaramillo Escobar, Daniela Giraldo Campillo, Karen Reyes Romero, Miguel A Neira Rincón, Mateo Zuluaga, Carlos M Ardila

**Affiliations:** 1 Department of General Surgery, University of Antioquia, Medellín, COL; 2 Department of General Surgery, Hospital Alma Máter de Antioquia, University of Antioquia, Medellín, COL; 3 Department of Emergency Medicine, Universidad Pontificia Bolivariana, Medellin, COL; 4 Department of Basic Sciences, Faculty of Dentistry, University of Antioquia, Medellin, COL

**Keywords:** cocaine, conservative treatment, mediastinal emphysema, pneumomediastinum, subcutaneous emphysema

## Abstract

Spontaneous pneumomediastinum (SPM) is a rare clinical entity typically associated with underlying pulmonary disease or trauma. However, it has also been linked to illicit drug use, with cocaine being one of the most common. We present the case of a previously healthy 23-year-old patient who arrived at the emergency department with retrosternal pain, odynophagia, dyspnea, and crepitus in the neck and chest after inhaling cocaine for three consecutive days. The patient was hemodynamically stable, with extensive subcutaneous emphysema in the neck and chest. Computed tomography of the neck and chest revealed abundant air dissecting the superficial and deep planes of the neck and mediastinum, particularly in its upper and middle portions. Additional studies included nasolaryngoscopy, which showed white material suggestive of inhaled substance use. An upper gastrointestinal endoscopy was performed to rule out perforation, which did not identify any lesions. Blood tests showed no abnormalities. Conservative management with analgesia and monitoring was initiated, resulting in the improvement of subcutaneous emphysema and pain. The patient was discharged after a two-day hospital stay. There were no complications or further visits to the institution within the following six months. This case highlights the importance of investigating a history of illicit drug use, particularly cocaine, in cases of spontaneous pneumomediastinum. Our findings support the generally benign course of this condition and the effectiveness of conservative management in the absence of complications.

## Introduction

Pneumomediastinum, characterized by air in the mediastinum, is a rare and intriguing clinical entity [1]. Typically, this condition arises from traumatic injuries, pulmonary diseases, or iatrogenic complications secondary to medical procedures. Spontaneous pneumomediastinum (SPM) can sometimes be diagnosed even when a clear etiology is absent. SPM is an uncommon phenomenon [2]. Notably, recreational substance use, particularly cocaine inhalation, has emerged as a significant risk factor for SPM [3].

The diagnosis of SPM poses a clinical dilemma, as it often presents with nonspecific symptoms, such as chest pain, dyspnea, and odynophagia. A thorough understanding of the typically benign natural history of SPM is crucial to avoid unnecessary diagnostic testing, invasive procedures, and associated complications. Prompt recognition and conservative management can significantly improve patient outcomes. This case report highlights the association between cocaine consumption and SPM, underscoring the importance of eliciting a thorough substance use history in patients presenting with spontaneous pneumomediastinum.

## Case presentation

A 23-year-old male presented to the emergency department with a two-day history of retrosternal chest pain, odynophagia, dyspnea, and subcutaneous crepitus in the neck and chest, following three days of recreational cocaine inhalation.

Physical examination revealed extensive subcutaneous emphysema in the anterior and posterior neck triangles, with bilateral and anterior emphysema extending to the anterior thorax, predominantly on the right side. Notably, there were no inflammatory changes or skin lesions. Laboratory studies (Table 1) demonstrated a slight elevation of C-reactive protein (CRP), with no other significant abnormalities.

**Table 1:** Laboratory results.

Parameter	Result	Units	Reference range	Units
Sodium	138.81	mmol/L	135-145	mmol/L
Potassium	4.65	mmol/L	3.5-4.5	mmol/L
Chloride	106	mmol/L	99-109	mmol/L
C-reactive protein	5.12	mg/dL	-	mg/dL
White blood cells	5440	cells/µL	4500-11,000	cells/µL
Neutrophils count	3670	cells/µL	1500-4000	cells/µL
Neutrophils percentage	67.5	%	50-70	%
Lymphocytes count	1280	cells/µL	1500-4000	cells/µL
Lymphocytes percentage	23.5	%	18-42	%
Hemoglobin	14.05	g/dL	-	g/dL
Hematocrit	40.1	%	40-54	%
Platelet count	187,000	cells/µL	150,000-450,000	cells/µL
Erythrocyte sedimentation rate	16	mm/hour	0-20	mm/hour

**Figure 1:**
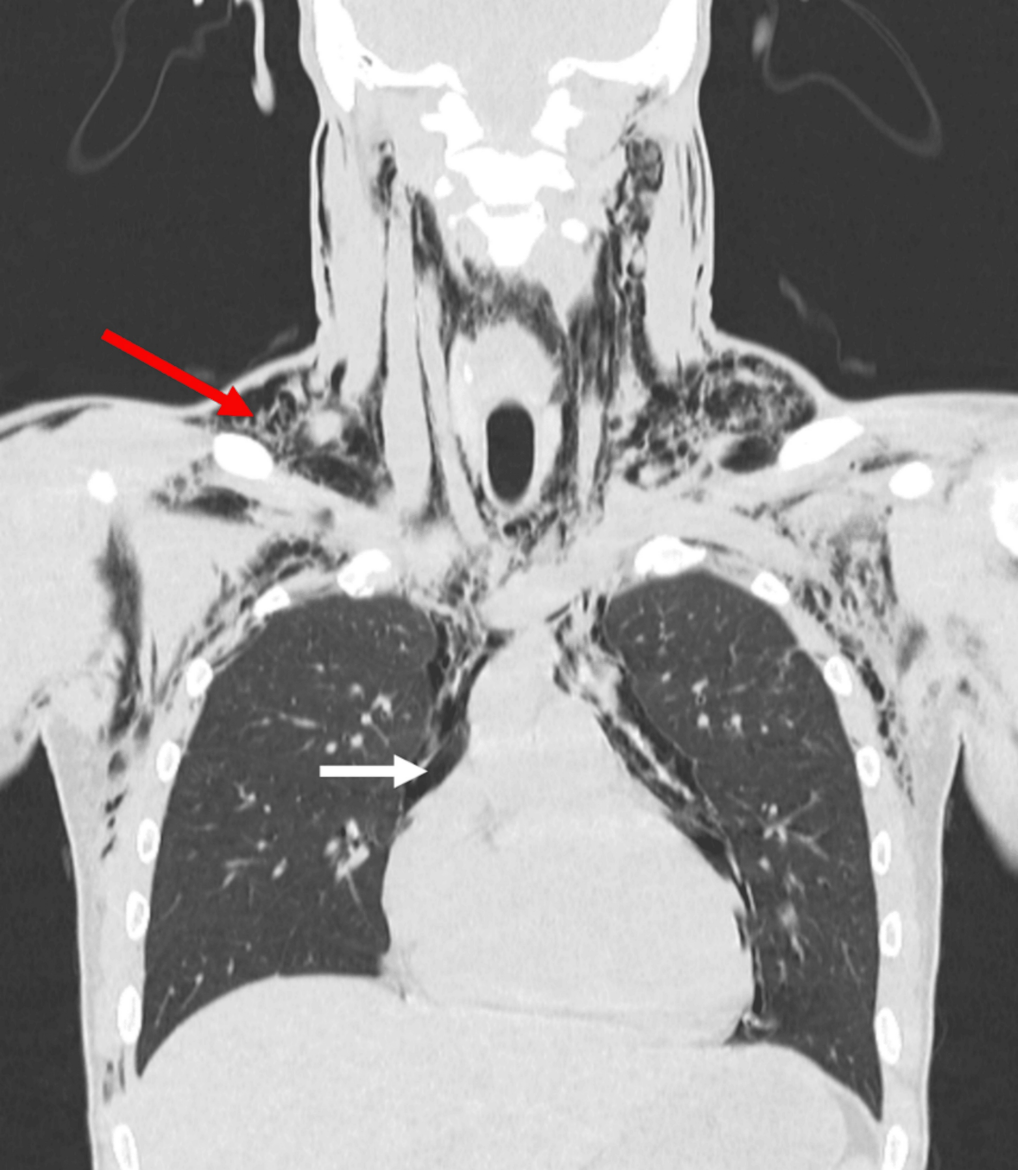
Coronal CT scan showing extensive subcutaneous emphysema in the neck and chest (red arrow) and significant pneumomediastinum (white arrow). CT: computed tomography.

Diagnostic imaging, including computed tomography (CT) scans of the chest and neck (Figure 1), revealed abundant air dissecting the superficial and deep planes of the neck, surrounding the thyroid gland and carotid sheath structures.

The air extended inferiorly into the thoracic wall muscles and dorsal region, dissecting the superior and middle mediastinum vessels, reaching the axillary region, and surrounding the trachea, aortic arch, pulmonary artery, and cardiac cavities without involving the pericardial space. Key findings included pneumomediastinum with abundant air in the superior and middle mediastinum, without evidence of collections.

Nasolaryngoscopy demonstrated chronic rhinitis changes, including whitish material adhered to the left nasal septum, turbinate hypertrophy, and a small dark plaque on the anterior wall of the left pyriform sinus, suggestive of inhaled substance use. Otorhinolaryngology evaluation ruled out any conditions requiring intervention.

**Figure 2:**
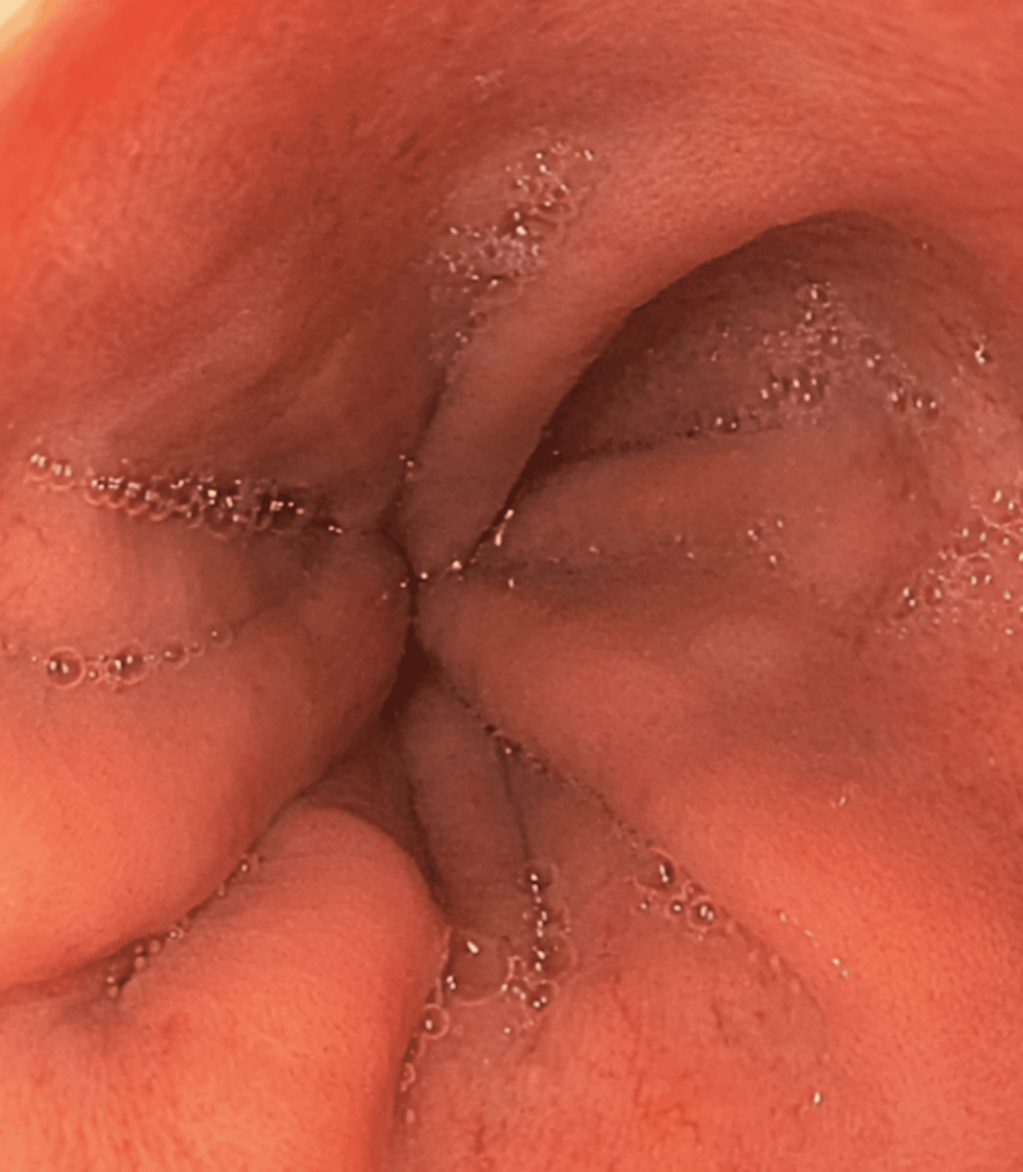
Image of the distal esophagus without mucosal lesions and wall defects or abnormal findings.

Upper gastrointestinal endoscopy (Figure 2) was performed to exclude esophageal perforation, revealing normal mucosa, caliber, and peristalsis.

Esophagoscopy, gastroscopy, and duodenoscopy showed no abnormalities. The patient was managed conservatively with clinical monitoring and analgesia, without invasive interventions. Outpatient follow-up demonstrated progressive improvement of subcutaneous emphysema, with no new symptoms. The patient was discharged after a two-day hospital stay with a favorable outcome.

## Discussion

Pneumomediastinum, characterized by air in the mediastinum, typically raises concerns about underlying serious conditions, such as mediastinitis or aerodigestive perforation. However, it can also present benignly, particularly in association with substance use, including cocaine [2-4]. The literature has reported this rare complication with diverse clinical presentations and underlying mechanisms.

The concept of pneumomediastinum was first described by Louis Hamman in 1938 [2]. When no apparent cause is identified, it is termed SPM. The pathophysiological explanation, proposed by Macklin in 1944 [3], involves the rupture of terminal alveoli in the pulmonary interstitium due to acute high intrathoracic pressure, known as the Macklin effect. This pressure difference facilitates air dissemination along vascular and fascial planes. An alternative explanation suggests the direct toxic effect of cocaine on lung tissue, leading to alveolar rupture [4].

Diagnostic imaging, typically initiated with chest radiography [1,5], is often supplemented with CT scans to provide detailed characterization [5]. When esophageal perforation is suspected, contrast studies or upper endoscopy are indicated to rule out serious secondary causes. However, in cases with a history of cocaine inhalation, these studies may be omitted, reducing healthcare costs.

In our case, the CT scan revealed extensive subcutaneous emphysema and pneumomediastinum. Additional studies, including endoscopy, ruled out other causes, confirming the relatively benign course of this condition [3]. Management of pneumomediastinum associated with psychoactive substance use, particularly cocaine, is generally conservative and effective [6]. Analgesics were sufficient to improve the clinical picture and facilitate patient discharge. Some studies suggest the use of prophylactic antibiotics and supplemental oxygen to aid in pneumomediastinum resolution [4]. Surgery is reserved for cases with esophageal perforation or pneumothorax requiring thoracostomy.

The literature indicates a favorable prognosis in most cases [3,4,7,8], supported by our findings. Recognizing this causal relationship can minimize unnecessary diagnostic testing and reduce healthcare costs.

## Conclusions

Cocaine-associated pneumomediastinum is a rare clinical entity with an extremely low incidence. Typical presentations include pleuritic chest pain, subcutaneous emphysema, and occasional dyspnea. While chest radiography may detect mediastinal air, CT provides superior characterization and is essential for confirming the diagnosis. A thorough history of cocaine use, combined with CT-confirmed pneumomediastinum, often suffices for management, which is generally conservative. Additional diagnostic studies, such as nasolaryngoscopy or endoscopy, are typically unnecessary, as most cases yield normal results. The well-described pathophysiological explanation of pneumomediastinum, involving alveolar rupture and air dissemination, supports this approach.
